# ‘Singapore on Thames’ Metropolitan Dreams and Planning Regimes

**DOI:** 10.1093/ojls/gqag008

**Published:** 2026-02-12

**Authors:** Justin Lim

**Keywords:** administrative law, environmental law, planning law, UK, legal culture

## Abstract

Singapore has frequently been idealised as a metropolitan financial hub that the UK should aspire to by adopting similar models of deregulation and executive control. This article brings attention to the role of planning laws in enabling or obstructing this goal. Planning regimes are responsible for facilitating a city’s infrastructural transformation, but are also emblematic of a state’s legal approach to its political goals. Comparing the planning regimes of the UK and Singapore will reveal how, despite originating from similar systems, their modern forms create vastly different pathways towards their metropolitan dreams. ‘Singapore on Thames’ reform in the planning context must thus be questioned as the legal histories and frameworks of the planning regimes also speak to irreconcilable legal cultures. In particular, differences in accountability mechanisms, the ways political authorities and their laws are legitimated and the role of law should warn against simplistic calls for deregulation or technocratic executive control. This comparative study of English and Singaporean planning regimes thus sheds light on how divergent approaches to planning may secure different outcomes, warns against legal reform that fails to account for the features of local legal culture and concludes by rethinking the concept of ‘Singapore on Thames’.

## 1. Introduction

‘Singapore on Thames’ is both a political strategy and a slogan. As a political strategy, it refers to the aim of promoting growth in the UK by transforming England into a ‘freewheeling, low tax, deregulated economy on the EU’s doorstep’.^1^ As a slogan, it evokes the image of Singapore’s metropolitan cityscape as a competitive global financial hub in the region—a ‘hi-tech, consumer-oriented kind of future of designer heels stepping into driverless cars’.^2^ Put together, politicians^3^ and commentators^4^ have both suggested that it represents the UK’s desire to rely on financial deregulation to achieve Singapore’s success as a trade and financial centre underpinned by political and social stability. But while some commentators have been quick to point out how the different political landscapes and freedoms between both states might make the adoption of Singapore-style regulation difficult in the UK,^5^ little to no attention has been paid to the role of planning regimes in facilitating the UK’s transformation into a ‘Singapore on Thames’.

Infrastructure plays a central role in establishing any financial hub or metropolis—a matter which Singapore recognises.^6^ By extension, planning regimes are critical in ensuring labour and business activities can run smoothly. Airports, housing, technological infrastructure and amenities all factor into whether a city is able to act as a hub for businesses, and if it can stay desirable as one.^7^ It is thus no surprise that the UK’s pursuit of Singapore-style financial deregulation coincided with ambitious planning reforms during the post-Brexit leadership of the Conservative Party.^8^ In order to ‘Build, Build, Build’, the Johnson government also sought Singapore-style reform of the UK planning regime: deregulation in the form of streamlining existing regulatory processes to expedite projects, and greater executive control through the introduction of zoning.^9^ Although the Johnson government failed to enact many of its ambitious reforms, much of the spirit of deregulating the planning regime and introducing more executive control lives on in the Labour government’s Planning and Infrastructure Bill 2025.^10^ Clearly, the desire to unshackle the UK planning regime from its regulatory controls is something more persistent than a political fad.

This persistent trend towards deregulation and executive control in the planning regime, which mirrors that of its financial law counterparts, thus demands greater scrutiny. While few have managed to draw parallels between the UK’s planning reforms to Singapore’s existing regime,^11^ the comparison is a lucid one. Singapore boasts a comprehensive planning regime where the planning agenda is centralised in the executive together with its administrative agencies, and where avenues for public participation or impact assessments are limited in order to facilitate the timely completion of development projects.^12^ But the success of its planning regime cannot be understood in isolation from its unique political and legal landscape, nor is the regime without its faults. The way the state wields the law to achieve planning outcomes must thus be sensitive to its own legal culture and circumstances.

Two questions are thus posed. First, how do the planning regimes of the UK and Singapore facilitate their similar metropolitan dreams? By focusing on the role that legal instruments and institutions play in each jurisdiction,^13^ it is argued that, while the Singaporean planning regime seems to allow development projects to be completed in a more straightforward and fast manner, legal and political features extraneous to the planning regime are significant factors in enabling this. In particular, the general culture of acquiescence or trust in a ‘technocratic’ government has resulted in relatively little challenge to development projects.^14^ The lack of actual legal avenues to raise such challenges further compounds this phenomenon, with recent controversies being resolved through non-legal channels. This model of Singaporean efficiency thus operates in a context where the frequency of conflict is low,^15^ and there exists limited but somewhat effective avenues for redress. In contrast, the UK planning regime has traditionally worked to provide avenues for participation and dispute resolution, which results in a deliberate and deliberatively democratic pursuit of development.^16^ But the different political and legal contexts of both jurisdictions mean that adopting Singapore-style reform will not necessarily allow the UK to achieve its metropolitan dreams with greater ease.

Second, what do the respective planning regimes tell us about the normativity of how each state achieves its planning objectives? Such a question is crucial to appreciating how a legal culture is identified and operates, thereby challenging how a transfer of laws without an understanding of institutional and social behaviour risks unnecessary disruption for governance.^17^ The concept of legal culture grounds the earlier comparative analysis by establishing the common functional concepts of which ‘law’ ought to comprise.^18^ In this sense, accountability, legitimacy and the role and rule of law are all concepts that make up the legal cultures of the UK and Singapore. These three features are undoubtedly dimensions of good administration, which the UK^19^ and Singapore^20^ courts have expressly described as features of the legal landscape. The discussion will demonstrate how it is unclear whether the UK or the Singaporean regime is normatively more appealing, as each can only be assessed within its specific legal culture.^21^

In answering both these questions, the article’s contribution to the literature is threefold. First, in performing a deep comparative analysis of how the planning regimes of the UK and Singapore operate, it contributes to the limited body of scholarship on the modern planning regimes of both^22^—particularly from a legal perspective, which has often been neglected.^23^ Second, the article’s arguments address the temptation to pursue deregulatory agendas felt across the world. These agendas take multiple forms: from Trumpian repeals of public participation avenues^24^ to an ‘abundance agenda’ that prioritises executive-led ambition over administrative intervention;^25^ elsewhere, the superiority of a ‘technocratic’ executive also presents similar promises of a blank cheque for governments to pursue their development agendas.^26^ Against these ideals, this article warns against blind acceptance that deregulation or greater executive control is the key to achieve growth or a ‘Singapore on Thames’.^27^ Finally, this article is an example of scholarship that is parochial—ie grounded in one legal context at a particular point in time.^28^ It challenges the seemingly universalist appeal of deregulation and executive control by demonstrating how legal frameworks and institutions ingrain themselves in their particular contexts, thereby casting doubt over whether the executive can truly address the vacuum left by deregulation.^29^ The institutional and functional comparative analysis of the UK and Singapore’s planning regimes may not provide answers for how to create a ‘Singapore on Thames’, but it may clue policy makers into an agenda for what sort of reform could work.^30^

To explore these themes and answer the questions posed in relation to the role of planning regimes, the article starts by explaining how ‘Singapore on Thames’ describes the common urban development goals for the UK and Singapore, and hence why their planning regimes should be scrutinised (section 2). Section 3 then discusses how their respective planning regimes enable or frustrate these metropolitan dreams. The purported obstacles that UK planning legislation poses is contrasted against the relative lack of legislation in Singapore, where a policy- and administration-centric approach has led to successful urban development. The comparison will challenge whether the recently proposed reforms to the UK planning regime are appropriate, let alone effective. In section 4, the respective legal cultures of the UK and Singapore are drawn out from the comparative study by thinking about the ways their planning regimes ensure accountability and legitimate development outcomes, and are supported by the role and rule of law. It is argued that any prospect of deregulation cannot ignore these themes as they risk overlooking various interests and resources necessary for the functioning of the planning regime. The article concludes by framing both planning regimes within the wider role and rule of law.

## 2. ‘Singapore on Thames’ as a Rallying Cry

‘Singapore on Thames’ was not originally a slogan used to describe the UK’s proposed planning reforms. This section thus starts by setting out the history of the slogan, before discussing how the metropolitan dreams underlying ‘Singapore on Thames’ apply to both the UK and Singapore. The discussion then considers the role of planning regimes in achieving these metropolitan dreams.

The term ‘Singapore on Thames’ was coined by the press in the aftermath of an interview with then-Chancellor of the Exchequer Phillip Hammond, which warned that the UK may have to change its economic model away from European-style economic regulation.^31^ In the wake of Brexit, one alternative to the existing state of affairs was to no longer comply with European regulations across multiple issues, like tax, financial services, labour and the environment. To that effect, one Conservative Lord suggested the UK could become ‘super-duper Singapore’ by removing ‘safety belt upon safety belt’ of EU regulation.^32^ Such proposals were certainly high on the ‘Leave’ agenda during the Brexit campaign, but ultimately petered out as they were seen as too radical a regulatory shift and detrimental for environmental protection and workers’ rights.^33^

However, ‘Singapore on Thames’ was not just a creature of Brexit. Tensions between market intervention and *laissez-faire* economics have existed throughout much of modern history.^34^ Pre-Brexit, this tension took the form of Euroscepticism in the UK, which was both a school of thought and a political movement to oppose the European integration project.^35^ Recent campaigns for deregulation or greater executive control relate to a host of factors: the increasing income inequality that has persisted throughout an era of globalisation;^36^ the ‘death of expertise’ of administrative agencies;^37^ and the populist appeal of a ‘strong man’ executive who can deliver solutions.^38^

Some of these ideas and issues resonate with Singapore-style governance, where a single political party that has controlled the executive since the country’s independence has charted the country’s success as a metropolitan global financial hub by embracing financial liberalisation.^39^ But this does not tell a complete story of Singapore’s success, nor is it the only way to achieve a state’s metropolitan dreams. After all, few would dispute that London remains one of the world’s leading financial centres, yet its path to success has been vastly different from that of Singapore.

One important way the paths of Singapore and the UK have diverged is in their planning regime, which influences each country’s ability to attract human capital and business activity by offering ‘world-class infrastructure’.^40^ The role of physical infrastructure and the standard of living have merely been recognised in the literature on the success of both countries as financial hubs,^41^ but have hardly ever been closely studied.^42^ This is despite how both countries recognise the role that physical infrastructure plays in building a strong economy. For Singapore, one of the key themes to its 2019 Master Plan is ‘Local Hubs, Global Gateways: Forging a stronger economy for all Singaporeans involves planning and setting aside suitable land to support efforts in rejuvenating our existing industries and the development of new areas of growth’.^43^ Similarly, the UK government has expressly recognised that ‘a failure to build enough critical infrastructure … is constraining economic growth and undermining our energy security’.^44^ The point is made clearer when one considers both countries’ experiences with executive-led proposals for airport expansion—a matter considered in more detail in section 3. To summarise: the expansion of Heathrow’s third runway was first raised in 2003, but has been beset by political and legal challenges so has yet to be built as of the time of writing.^45^ In contrast, the expansion of Singapore’s Changi Airport has been largely unfettered, with two new terminals and a 10-storey luxury mall annexed to the terminals built within the same period, as well as plans to build yet another terminal in the next decade.^46^ The UK government’s experience with the Heathrow expansion thus speaks to their wider concern that a great delay to nationally significant infrastructure projects (NSIPs) may hinder their overall growth strategy.

Parallels can thus be drawn between the story of economic slowdown and development struggles of both countries. ‘Singapore on Thames’ is an ideal that captures the continued economic and development success that Singapore has enjoyed—the idea of Singapore ‘exceptionalism’.^47^ Across both financial and planning regulatory regimes, Singapore-style executive-led decision making with minimal legal intervention is seemingly responsible for its exceptional success. While the UK may not be expressly modelling its planning reforms with reference to Singapore, its desire to streamline planning by limiting existing channels of participation, restricting review of development consent and strengthening the role of executive-led planning strikes multiple chords with Singapore’s existing planning regime. This is echoed in the deregulatory ethos of the ‘Abundance’ agenda, which proposes that environmental legislation and litigation, rather than political division, are the primary obstacles to building cities that serve as centres of power and wealth.^48^ Rather than a policy position, ‘Singapore on Thames’ thus works as a slogan that embodies the spirit of the UK’s planning ideals and reforms. Section 3 thus turns to how the planning regimes of both countries enable and frustrate their common metropolitan dreams that underscore the concept of ‘Singapore on Thames’.

## 3. How Planning Regimes Enable and Frustrate Metropolitan Dreams

This section explores how the UK and Singapore planning regimes operate to achieve their similar metropolitan goals. However, space precludes a complete canvas of their entire planning regimes, let alone the regulations for specific types of buildings, like housing. Rather, the discussion focuses on three core aspects of the planning regime in relation to NSIPs.^49^ First, the legislative and policy frameworks for development planning will be scrutinised. Second, the ways in which those frameworks interact with public participation and judicial review will be considered. Finally, the role of the executive and its administrative agencies will be emphasised, particularly since the deregulatory agenda discussed in this article generally grants the executive greater decision-making power relative to the public and the judiciary.

Across these themes, differences between the UK and Singapore’s planning regimes and legal cultures will reveal why the Singaporean planning regime might stand for the proposition that an executive-led system with minimal democratic involvement in decision making best facilitates development objectives. The section then concludes by considering whether the UK’s proposed reforms, which resemble a convergence between the two planning regimes, will actually allow it to fulfil its metropolitan dreams to become a ‘Singapore on Thames’.

### A. UK Planning Regime

English planning law has consistently featured multiple tensions that also shape the way we think about its pursuit of ‘Singapore on Thames’ today. These tensions are instructive in understanding how decisions are made and why there may be delays in achieving development goals. The first group of tensions relate to centralised versus non-centralised planning, which reflects ongoing debates on whether planning law should take a comprehensive or incremental approach, or whether it should necessarily take a top-down approach.^50^ The second group of tensions refer to the tripartite interests of public interest as defined by the executive, the private property interest of the landowner and public participation in the decision-making process.^51^ These dimensions relate to the polycentricity of environmental problems more generally, where decision making is especially difficult as it can have unintended effects on multiple parties, thereby creating multiple ‘losers’.^52^ Finally, the last tension has been especially pronounced in a post-Brexit world: the role of domestic legislation versus EU regulation. In particular, the implementation of the EU’s Environmental Impact Assessment Directive^53^ and Strategic Environmental Assessment Directive^54^ has significantly influenced the UK planning regime and jurisprudence on planning decisions.

These tensions have ebbed and flowed in the minds of policy makers and the public throughout history, resulting in a relatively fragmented planning regime. The task for planning decision makers, courts and lawyers is thus to appreciate how these tensions sound out in the legal and policy frameworks. This is not an easy task. To start with, the ‘plans’ of the planning regime are numerous, and can all play a role in determining whether a particular development should receive planning permission or development consent. First, local authorities have a duty to create a ‘local development framework’ under section 13(1) of the Planning and Compulsory Purchase Act 2004. The local development framework comprises a portfolio of local development documents, like the overall strategy for the locale, site allocation proposals, area-specific action plans and a statement of community involvement.^55^ These documents are generally prepared by the local planning authorities, and consider a range of issues, like climate change, housing and industry, across a 15-year timeline.^56^

Second, the local development frameworks are made relevant for the decision making of all planning authorities through the non-statutory National Planning Policy Framework (NPPF), which is a comprehensive policy document prepared by the government to indicate the considerations planning authorities should take into account when granting planning permission.^57^ The NPPF also stipulates how other statutory plans are to be considered, such as the spatial development strategy prepared under the Greater London Area Act 1999 or neighbourhood plans under the Localism Act 2011. Third, planning practice guidance comprises *ad hoc* emanations of central government policy that hold similar status to the NPPF. Unlike the NPPF, they are not comprehensive policy documents, but usually aim to ‘amplify published policy’ or clarify certain policies in the NPPF.^58^

Relatedly, the fourth planning document is the National Policy Statement, which is prepared by the relevant government departments pursuant to section 5 of the Planning Act 2008. Unlike the NPPF, National Policy Statements specifically provide for the proposals and considerations in relation to NSIPs. However, this is not to say the NPPF and local plans become irrelevant where NSIPs are concerned. The NPPF may clarify certain definitions of terms in the National Policy Statement, local development framework or statute. For instance, the UK Supreme Court referred to the NPPF’s explanation of the objective of sustainable development to clarify the Secretary of State’s duty to ‘[contribute] to the achievement of sustainable development’ when preparing National Policy Statements.^59^ Furthermore, whether development consent orders are granted for NSIPs pursuant to a National Policy Statement can also bear on local development frameworks and the NPPF as ‘any matters prescribed in relation to development’ and ‘any other matters which the Secretary of State thinks are both important and relevant to the Secretary of State’s decision’.^60^ However, it is noted that the White Paper preceding the Planning Act 2008 had envisioned National Planning Statements to have more weight than other planning documents^61^—but this intention was not ultimately legislated into the Planning Act 2008.

Finally, perhaps in response to the lack of hierarchy between local plans and those prepared by the government, the Levelling Up and Regeneration Act 2023 amends the Planning and Compulsory Purchase Act 2004 to introduce a National Development Management Policy (NDMP).^62^ This policy has equal weight to local development frameworks, but will take precedence over local plans in the event of conflict.^63^ However, the statute does not specify what this policy is supposed to contain, only that the Secretary of State must have regard to climate mitigation and adaptation.^64^ There is also no published instance of an NDMP, yet even more new types of plans are proposed under the Planning and Infrastructure Bill 2025 (discussed in section 3C). Given that there is no duty for the Secretary of State to prepare an NDMP and that the Levelling Up and Regeneration Act 2023 was a watered-down version of the Johnson government’s proposed planning reforms, it remains to be seen if any new plans or policies will be designated as an NDMP.

Taken all together, there are thus multiple statutory and non-statutory mechanisms by which the central government can introduce their plans for development. Each series of statutory and policy reforms seeks to reduce delay in achieving ‘Singapore on Thames’, but Marshall and Cowell observe that these interventions do not actually ‘accelerate’ project timelines.^65^ Underlying each planning policy document is a reconfiguration of the planning decision-making process, which merely reallocates the delay at one stage to another. Furthermore, these reforms do not shield both plans and planning decisions from public scrutiny and judicial review.

Public consultation and judicial review are deeply embedded in the planning practices of the UK. Plans prepared pursuant to statute will generally include provisions that the public be consulted when preparing such plans, and that such plans may also be subject to judicial review.^66^ In the NSIP context, an ‘inquisitorial’ examination is undertaken where local peoples can make written representations at the pre-application stages of planning.^67^ In addition, public consultations are part of the strategic environmental assessment before any plan or policy is adopted.^68^ Similarly, prior to the grant of planning permission or development consent, developers must perform an environmental impact assessment, which also requires consultation with the public on the impact the development may have on their lives.^69^ In relation to judicial review, even without statutory intervention, because plans are types of policies, they are legal instruments that may be scrutinised by the courts for interpretation and to construe their legal effect.^70^ Statute also provides that planning permission and development consent orders may also be judicially reviewed.^71^

The interface between the participatory avenues within the planning regime and the judicial reviewability of plans and decisions has two important consequences for the way the planning regime may achieve the central government’s metropolitan dreams. First, and quite obviously, public consultations take time, and challenges to decisions may cause further delay. The government has not shied away from pointing out how ‘delays to these major projects have serious implications, including holding back the delivery of essential benefits to the country and imposing considerable additional costs on development’.^72^ The aforementioned difficulties in building the third Heathrow runway best exemplifies this: over two decades of planning and review, and still the runway remains incomplete.

The litigation born out of the Heathrow saga demonstrates how the normative tensions in the planning regime play out between administrative agencies, the courts and lawyers. To set out the brief facts of the case, the litigation was commenced by a group of environmental non-governmental organisations that sought to challenge the government’s Airport National Policy Statement, prepared under the Planning Act 2008, which contained proposals to expand Heathrow airport. The claimants contended that the Statement was unlawfully prepared as it failed to take into consideration the UK’s commitments under the Paris Agreement as a material consideration and, per the government’s obligations under the Environmental Assessment of Plans and Programmes Regulations 2004, did not ‘contribute to the achievement of sustainable development’ per section 10 of the Act, nor did it consider the impacts of non-carbon dioxide emissions. While the Court of Appeal found for the claimants on the ground that the Paris Agreement was a material consideration that the government had failed to consider,^73^ that decision was overturned by the Supreme Court, which clarified that the ‘policies’ the government had to consider under section 5(8) of the Planning Act had to be given a narrow meaning. Government policies had to be ‘unambiguous and devoid of relevant qualification’, and the Paris Agreement was no such policy.^74^ Furthermore, the Supreme Court reasoned that the government, in considering its obligations under the Climate Change Act 2008, would have considered its obligations under the Paris Agreement.

The *Heathrow* litigation exemplifies how politically challenging and scientifically technical planning decisions, when made under a complex legal framework that does not prescribe clear positions on important normative tensions, require rational explanations and clarifications of the law in order for particular planning outcomes to be achieved. Three observations can be made in relation to these tensions. First, the *Heathrow* litigation was essentially a testing ground for the National Policy Statement regime for NSIPs under the Planning Act 2008. While the regime was originally envisioned as a way to expedite NSIPs through executive-led planning,^75^ the extensive public consultations, political inquiries and litigation demonstrates that complex environmental decision making cannot be so easily streamlined. Second, the litigation demonstrates how regional and international legal instruments also factor into the way planning decisions are made. This is despite the fact that the Paris Agreement was not directly enforceable in the UK courts. The public was still interested in how the government would fulfil its obligations under the Paris Agreement, which then became a matter for the courts to determine if this was a material consideration for the preparation of the Airport National Policy Statement. Finally, the tensions between democratic input and the effectiveness of the planning regime were on full display. Not only had there been public inquiries, consultations and further reviews by independent committees like the Committee on Climate Change, it was clear that some members of the public were still dissatisfied with the Airport National Policy Statement and wished to challenge the proposals through judicial review.^76^ This is not to say that public participation channels are unproductive for planning regimes, but rather it raises questions of the way public consultations are conducted, alongside how feedback is taken into account and responded to.

Decision makers must anticipate and address these tensions in order to prevent disproportionate delay. But decision makers, in the form of the executive and its administration, are not uniform or static entities. The privatisation of public functions and funding in the late 2000s introduce new interests into the planning decision-making matrix. For instance, Marshall points out how the Civil Aviation Authority had ‘intervened to dramatic effect after 2008, to force the [British Airports Authority] to sell two of its three London airports’, which sets the stage for the eventual *Heathtrow* litigation.^77^ Furthermore, the political, and hence development, agenda changes are liable to change between electoral cycles and political controversies. Thus, it is not only the public and the judiciary that seem to be delaying planning decisions; the diverse and dynamic interests within political and administrative institutions achieve a similar effect.

The diverse interests and responsibilities across the executive are significant as they undermine the claim of ‘democracy by lawsuit’, where ‘legal thinking centers around statutory language and commitment to process, not results and outcomes’.^78^ It is clear from the English planning regime that planning decisions are not merely a binary matter of whether a project is built or not; rather, the way in which a project is conceived and built is of great importance. This is because planning is ‘inevitably, a sensitive process, since it constrains the freedom of private owners to use their own land as they wish’.^79^ What underpins the regime, and hence what the UK courts seek to uphold, are the ‘touchstones’ of ‘democracy, accountability, and review’.^80^ Courts, administrative agencies, the executive and the public form the basis of a deliberative democracy where a legal system strives towards reaching collective decisions to reflect the plural interests at play.^81^ The executive and its administration make decisions based on the relevant expertise and provide good reasons in doing so. The public will often only be heard if it can raise its concerns in a way that relates to this expertise. Courts then play a role in investigating whether this expertise is relevant and if the reasons given are lawful.^82^ These mechanisms form a system by which planning policies and decisions are meant to be reviewed before outcomes are secured, otherwise such outcomes may not be worth securing at all. The UK planning regime is thus deliberately deliberative. It places an emphasis on the way decisions are made, at the expense of relative delays in achieving metropolitan dreams like ‘Singapore on Thames’.

### B. Singapore Planning Regime

Having discussed how the UK’s planning regime operates to achieve metropolitan dreams, one might question why the conclusion that a planning regime is designed to ensure planning decisions are well reasoned is novel, let alone interesting. After all, is that not the point of public law,^83^ or any law?^84^ Arguably, Singapore’s planning regime challenges this view. An exploration of the relative lack of legal frameworks and judicialisation of the planning process, as well as the vastly different nature of its political institutions, positions the Singaporean planning regime as an alternative paradigm.

Despite inheriting a planning regime from the British colonial rule, Singapore’s modern planning regime has evolved far differently from the UK’s. Unlike the UK, there is only one statutory planning policy—the Master Plan, which must be reviewed once every five years.^85^ The Master Plan is essentially a zoning exercise led by the Urban Redevelopment Authority, as various areas are demarcated and the types of infrastructure that may be built in each area are specified.^86^ The conclusions of the Master Plan are informed by the non-statutory Long-Term Plan (previously called the Concept Plan), which dictates the 50-year outlook for Singapore’s infrastructure. The Long-Term Plan is both reviewed every 10 years and helmed by the Urban Redevelopment Authority,^87^ but is the result of multi-agency consultations.^88^ The coordination between agencies is evident in how planning objectives, like building more close-knit towns for housing areas, is present in both the Long-Term Plan and the strategies published on the relevant administrative agency’s website.^89^

Precise decisions on the particular development that may be built are then subjected to the development consent procedure. An application for development must be made by the owner of the land or with his consent.^90^ However, around 90% of land in Singapore is owned by the government, although most is held indirectly by the government through investment companies on a long lease.^91^ Approval for the planning permission has to come from the Urban Redevelopment Authority,^92^ and further clearance must be sought from the Frontline Technical Departments if the proposed development bears on matters like transport or the environment.^93^ Unlike in the UK, where planning applications feature multiple parties with different interests, much of the interaction between parties is understood in relation to the executive and the administrative bodies it has charged with particular domains (eg traffic, fire safety). Particularly in the case of NSIPs, it is also possible for the government to reacquire land, to gain further ownership over the development process, through the Land Acquisition Act 1966.

The executive thus has significant latitude in the planning regime: from charting the direction of development for the nation as a whole to planning for NSIPs, and finally by having access to the avenues for land acquisition to effect their plans. However, this is not to say that planning decisions are wholly a result of executive decision making as empowered by a closely aligned administration. There do exist channels for the public to raise their concerns in relation to the development plans and decisions. However, unlike in the UK, these channels are limited, and it is unclear how public feedback factors into the final decision. For instance, members of the public may submit objections to the Master Plan within two weeks of the proposed Master Plan’s publication.^94^ The statute then requires the relevant minister to invite the objector to be heard, or convene a public inquiry, unless they find the objection ‘frivolous’.^95^ The minister may also decline to publish the proposal for objections if they are satisfied that an amendment to the Master Plan is not ‘material in nature’.^96^ There is no obligation to publish the objections nor to address them comprehensively. Rather, such data must be requested of the minister or administrative agency, such as through parliamentary questioning. In 2014, the Minister for National Development responded to such a request in Parliament, sharing that the 2014 Master Plan received 402 pieces of written feedback, the feedback was incorporated where ‘feasible’ and that ‘one hearing’ was conducted. Relatedly, the Long-Term Plan also receives publicity and the public may provide their feedback. However, such consultations seem *ad hoc*: a ‘lifestyle survey of 4000 respondents’ was conducted in the 2011 and 2013 plan review;^97^ the ‘public engagement journey’ launched in 2021 is also *ad hoc* in the sense that its four phases across 2021–22 are organised and planned by the Urban Redevelopment Authority, where both administrative agencies and government representatives not only receive feedback but help the public understand ‘potential implications of each strategy, and the wide spectrum of competing needs for our limited land’.^98^ The government and its administration thus has significant discretion in how it chooses to receive, address and dispense with public feedback for development plans in the planning regime.

Unlike the opportunities to provide feedback on the plans of the planning regime, there are much fewer opportunities for the public to provide feedback on individual planning decisions. Unlike the UK, Singapore has no legislated Environmental Impact Assessment nor Strategic Environmental Assessment framework. Some NSIPs or developments may have an Environmental Impact Assessment performed where the government deems that such an assessment is necessary when the environmental impacts are deemed ‘significant’.^99^ Again, this means that the government gets to choose when and how public consultations are conducted for individual developments.

Yet the metropolitan landscape of Singapore, and few documented objections, seem to suggest that this is a planning regime that works to achieve great results. However, the minimal objections must be properly contextualised against Singapore’s political and legal backdrop. Four features of this backdrop explain why, compared to the UK, there is minimal criticism and hence delay in relation to development planning and consent. First, as explained, there are simply much fewer formal avenues within the planning regime for the public to raise objections. Second, compared to more liberal democracies, there is no free right of assembly or protest in Singapore. Those who wish to demonstrate must apply for a permit and may only do so at one designated location.^100^ Third, there is minimal judicialisation of governance where tribunals or courts supervise the regulatory functions of administrative bodies so that standards are set, processes are monitored and judicial decisions are enforced.^101^ Standing requirements for public interest litigation for breaches of constitutional rights remain strict: the applicant must demonstrate that his constitutional rights have been violated and ‘special damage’ has been caused,^102^ or that the state has breached a public duty in ‘such an egregious manner that the courts are satisfied that it would be in the public interest to hear’.^103^ These standing requirements complicate matters for litigants who may only suffer harm in the future or are concerned about the environmental impact of development. Claimants impacted by planning decisions may find relief in the fact that these standing requirements may not extend to administrative decisions.^104^ However, if the planning decision has been referred to and decided by the minister, it is ‘final’, so no further appeal to the minister or challenge through the courts is permitted.^105^ With few statutory procedural frameworks to hold the government and its administrative agencies accountable to, a political atmosphere that limits free protest and strict standing requirements, it is no surprise that there has been little litigation in respect of the planning regime.

Fourth, where planning decisions are litigated, the courts are ready to defer to the executive and its administration. The courts have found the administrative agencies to be acting within their discretion on polycentric issues of land valuation and planning, and will be slow to review such cases.^106^ A significant pattern to the already scant litigation in this respect is how there is little procedural legislation that dictates how administrative agencies ought to make decisions. For instance, the change in the land valuation method from what was previously represented by the Singapore Land Authority in a circular to a method that was never publicised was found to be within the discretion of the Authority as it was entitled to have ‘regard to efficiency and economy and to the social, industrial, commercial and economic needs of Singapore’ per section 6(2) of the Singapore Land Authority Act 2001.^107^

This is not to say that there ought to be more conflict within the Singaporean planning regime. It cannot be disputed that the executive-centric regime has frequently achieved its metropolitan dreams so that Singaporeans can enjoy a high-quality urban life. However, the point is that this is a regime that can achieve such results because of its political and legal context. The nascent culture of judicial review, combined with the relative political stability and suppression, mean that there are few stressors for the planning regime itself. This is evident from how commentators frequently credit Singapore’s built environment to Singapore’s political leadership.^108^ Just a year after independence, it was noted that recent leadership in the metropolitan planning programme ‘has come mainly from the political quarter. In close touch with the masses, it has formulated both bold and realistic development programmes to deal with the social and economic problems of the metropolis.’^109^ The public’s trust in the government’s steady hand has endured, with the ruling People’s Action Party consistently dominating at the polls, reflecting a general nationwide approval for its decisions.^110^ This combination of consistent executive-led decision making in respect of urban planning and infrastructure and the apparent high level of trust in the government has shaped the scholarly discussion around the Singapore planning regime. It is recognised that the administrative state of Singapore uses law as a facilitative tool to ‘[carry] out the country’s development plans and socio-economic programs’.^111^ Consequently, less attention has been paid to the ways in which such outcomes are arrived at.

Singapore’s political stability is not the only feature that sets it apart from the UK’s political context: there is also greater homogeneity and coordination between the Singaporean executive and its administration. It would not be a mistake to attribute both the Master Plan and the Long-Term Plan to the Singaporean central government, despite the fact that they are *prima facie* the creation and responsibility of its administrative agencies. Tensions arising from delegation, like those between administrative agencies and the central government, are minimal in Singapore. This is because the ruling party, the People’s Action Party, has governed Singapore since its independence, which means the civil service has thus only served one master throughout Singaporean history. Furthermore, there remains a close intellectual and political connection between the civil service and the People’s Action Party, as top civil servants are often recruited into the Party, thereby joining politics.^112^ The result is often a close synergy between the minds and expectations of the civil service and the executive, resulting in a ‘highly efficient’ civil service that can attend to the visions of the executive.^113^ This synergy means that plans between the government and its agencies are unlikely to be in conflict with one another, nor will developers or other administrative agencies need to balance between the considerations listed in different plans. Unlike the UK’s NPPF or Local Development Frameworks, the Master Plan already comes to a decision on the types of developments that may be built.

Despite the high incidence of inter-agency collaboration and trust in the executive, prolific objections to developments do still occur, but they are often voiced through the media and addressed through political statements. For example, when a Kranji woodland was mistakenly cleared by a developer, the Minister for National Development assured the public that a ‘framework to guide how and when environmental studies should be done’ would be considered.^114^ In the aftermath, the government assured that it would learn from its mistakes and better engage the environmental community. These are lessons for the exercise of the executive’s discretion which may create more processes or delays within the planning regime when planning processes within administrative agencies become more tedious. But they are not the same structural legal avenues for deliberation that may cause delay in the UK regime. When contrasting the two regimes, it thus seems like the UK’s planning regime resembles a winding path to its metropolitan dreams, whereas Singapore has maintained its highway towards urban success.

### C. Converging Paths to Metropolitan Dreams?

The pertinent question for the UK seems to be whether its winding path can be straightened. While some proposals, like the NDMP, resemble an attempt to build a highway over the winding road by creating a plan that should take precedence over other plans, such mechanisms are unlikely to succeed given the various other avenues by which policies and decisions may be challenged. The UK has tried to reform its planning regime to adopt deregulatory and centrally controlled elements we can observe in Singapore’s regime. But it cannot change the wider political and legal context its regime resides in, which means the existing normative tensions underscoring the planning regime would still have to be addressed under the new legal frameworks. Three areas of reform across the Levelling Up and Regeneration Act 2023 and the Planning and Infrastructure Bill 2025 illustrate this point.

To start with, the environmental outcome report regime of the 2023 Act provides that administrative agencies may make provisions to require an ‘Environmental Outcome Report’ prior to awarding planning permission or development consent.^115^ ‘Environmental outcomes’ are ‘outcomes relating to environmental protection’ specified under section 152 of the Act. The Environmental Outcome Report regime was intended to be a replacement of the existing Environmental Impact Assessment regime, which originated from EU law. However, because the old regime was not repealed, the Environmental Outcome Report regime is effectively a policy regime that runs in parallel with the existing legal Environmental Impact Assessment regime.^116^ Ogden-Jones observes that the Outcome Reports have a different normative basis from Impact Assessments as they are ‘more project-specific, considering whether environmental risks are proportional to the broader non-environmental objectives of the development, meaning [Outcome Reports] do not necessarily require a developer to consider all potential harms, as with the [Impact Assessments]’.^117^ The result is that, should an Environmental Outcome Report be required for developers, compliance with the regime is unlikely to make achieving development goals any easier. For one thing, the developer may still be in breach of their duty to prepare an Environmental Impact Assessment.^118^ For another, the Outcome Report cannot result in ‘environmental law providing an overall level of environmental protection that is less than that provided by environmental law at the time this Act is passed’.^119^ This means that developers effectively have double the regulatory cost in preparing two reports that serve the same purpose. Or, the Outcome Report may be challenged for resulting in a lower standard of environmental protection because it was prepared differently from an Environmental Impact Assessment.

The additional regulatory costs and likelihood of fresh challenges highlight the existing tension between domestic laws and regional regulations like those from the EU. EU regulations are sometimes viewed as expensive and time intensive, thereby causing inordinate delay in the planning regime.^120^ But such regulations have a purpose in ensuring certain levels of environmental protection and ensuring that planning decisions are rationally made.^121^ Where the planning regime has already been accustomed to certain types of reasoning, public participation and environmental consciousness, these expectations are likely to persist even upon removal of the whole assessment regime post-Brexit. These reasons also mean that the proposed ‘Environmental Delivery Plans’ under sections 53–6 of the Planning and Infrastructure Bill, which may be a third alternative or perhaps a replacement to the existing Environmental Impact Assessment and Environmental Outcome Report scheme, may not have the intended effect of expediting the planning process. The UK’s regulatory context is unlike the Singaporean context, where a requirement for impact assessments was never legislated, and there is relatively less attention paid to the planning decisions of the government. The persistent tensions between the regulatory purpose of EU policy and the national interests of streamlining the planning regime have made the alternative assessment regimes into an awkward piecemeal reform that does not really assist in achieving the UK’s metropolitan dreams.

Similarly, there is a greater frequency of executive-led plans and decisions. For one thing, there are proposals to introduce ‘call-in powers’ for ministers to prevent applications from being rejected by local councils.^122^ Similarly, Spatial Development Strategies introduce zoning and greater executive control of the planning agenda by specifying what may be built in particular areas at a sub-regional level.^123^ This seems to address the tension between local and executive-led planning, but the Planning and Infrastructure Bill does not actually state which plans take precedence or that local plans must be consistent with the new Spatial Development Strategies. In attempting to ‘strike a balance between providing strategic direction [and] the geographies in which people spend most of their lives’,^124^ this would only introduce more elements to the already complicated medley of planning policies and considerations for planning authorities. As with the Environmental Outcome Reports, the UK government is keenly aware it cannot totally shift towards a regime like Singapore’s, where all planning policies are harmonised and heavily determined by the executive. That would involve the repeal of multiple statutes, which would itself be a gargantuan and politically unpopular exercise. The result is that the tension between local and executive decision making might tip towards the policies of the executive, but the tension must continue to be clarified in planning documents, public consultations and before the courts—delays that the UK planning regime would have failed to avoid with its proposed reform.

Finally, the 2025 Bill proposes to limit permission to appeal decisions on NSIP planning policies and permission if the High Court decides that the application is ‘totally without merit’, and to make changes to the Civil Procedure Rules to decide permissions for appeal for judicial review at an oral hearing.^125^ By limiting the opportunities for review and providing speedier forums for deciding if judicial review should be allowed, this seems to bring the planning regime closer to Singapore’s legal landscape where judicial review of planning policies and decisions have been limited. However, it should be remembered that the Singapore political and legal landscape is one of disinterest, disincentive and disappointment before the courts in respect of planning policies and decisions. Singaporeans are generally disinterested in challenging planning decisions, politically disincentivised from doing so and likely to be disappointed if they do receive permission for judicial review. In contrast, while the proposed reform to limit judicial review avenues may indeed expedite planning decisions, it may ironically be contested on other grounds. For instance, Hawkins argues that these measures unduly restrict the ordinary citizen’s access to justice,^126^ which might lead to challenges for a breach of article 6 of the European Convention on Human Rights, which is given effect by the Human Rights Act 1998. Similarly, ‘removing the “paper permission” stage of judicial review’^127^ may lead to longer oral hearings as the overall review process becomes compressed, or to a greater volume of challenges at different stages of the planning process if the reformed judicial review procedure is perceived by claimants to be inadequate.

The UK’s attempts to streamline its planning regime to achieve its metropolitan dreams may thus fail to have its intended effect in the short term. This is not to suggest that more radical reform is necessary, but it draws into sharp relief the distinction between ‘Singapore’ and the ‘Thames’. The planning regimes of the two states continue to stand at opposite ends of the spectrum, where one exists in a system with few normative tensions, but the other has multiple competing tensions it must address at every stage of a comprehensive legal framework. Attempting to simplify or change the UK’s regime, to potentially enjoy the fruits of a less regulated executive-led regime like Singapore’s, is no simple task—‘what is needed [is] a change in political culture, not just a change in legislation’.^128^ As section 4 will discuss, this speaks to how planning regimes exist within their specific legal cultures, thereby stressing the importance in understanding the normative justifications for each regime. The difficulty of achieving ‘Singapore on Thames’ is thus not merely one of how planning regimes operate, but rather one of why planning regimes as legal devices exist in the first place.

## 4. Planning Regimes and Reform in Context

Thus far, this article has focused on the way the UK’s planning regime responds to its aspirations of ‘Singapore on Thames’. However, to appreciate how the English planning regime is situated in its legal context, a comparative study with Singapore is especially useful. By taking an institutional approach, the way accountability, legitimacy and the rule and role of law operate in the context of the two jurisdictions’ planning regime will be elucidated. These three dimensions are well understood to form part of a country’s legal culture. Legal culture, in turn, is a necessary lens through which prospects of deregulation are assessed.

Regulatory reform does not take place in a legal or political vacuum. This is particularly the case for planning law, which bears on national, sub-national and individual interests in the natural environment.^129^ Ideals of ‘abundance’ like Klein and Thompson’s sidelines this inherent complexity in planning regulation by characterising conflicting interests in decision making as obstacles to abundant outcomes.^130^ Yet, determining what is worth having in abundance is itself not a simple exercise, as decisions on infrastructure necessarily require a prioritisation of particular social goods and urban space. Planning is thus political. But the vehicle by which such politics are advanced takes the form of legal frameworks and procedural obligations that shape the way stakeholders’ interests are heard and evaluated. The proposed reform in the UK fails to acknowledge the non-legal challenges felt by different stakeholders to the planning process, preferring to instead frame these stakeholders as obstacles to planning delivery. Through the lens of legal culture, the ways legal reform speak to the enduring power structures and relationships of a jurisdiction’s administrative law are exposed.^131^ Analyses of public accountability, legitimacy and the rule and role of law can thus illuminate the extent to which, and why, ‘Singapore on Thames’ is mere fantasy for planning regimes.

### A. Accountability Mechanisms

A relationship of accountability refers to the ability of those who have a legitimate interest in a matter to hold the party responsible for the matter answerable for their actions.^132^ It is fundamental to any legal system that all are held accountable to the law. In a system of administrative law, which planning regimes reside in, decision makers must be held accountable for their decisions. However, the ways in which they are held accountable are not uniform. Distinctions may be made between holding them accountable for the way a decision is arrived at rather than the actual decision itself (procedural versus substantive), or to hold them accountable through legal channels like the courts rather than political channels like elections or consultations (legal versus political accountability).^133^ What decision makers are held accountable for, and how they are held accountable, can thus vary according to the legal regimes and cultures of the state.

This is also true of the UK and Singapore’s planning regimes. As discussed, the frequency and ways in which planning authorities are held accountable are vastly different. At first blush, it might seem like there is little desire to hold decision makers accountable under the Singapore regime, especially when compared to the vigour with which planning policies and decisions are litigated in the UK—particularly with the *Heathrow* saga. However, this fails to account for the multiple instances by which the Singaporean public voiced objections or concerns over development projects. In the late 1980s, the Nature Society had sought to influence government development decisions, such as by preparing their own Environmental Impact Assessment to object to the building of a golf course over a forest at Lower Peirce. Their 17,000-signature-strong campaign received mixed reception from the government, with the president of the Society receiving a call from the Internal Security Department.^134^ The decision to build the golf course was eventually called off. Since then, environmental interest groups like the Nature Society have increasingly been consulted when the government deems appropriate. This could involve consultations at the planning stage in closed-door talks, or after media attention and public debate.^135^ There is thus an appetite to challenge planning decisions through informal channels. However, the matter is trickier when development proceeds in unintended ways, such as when a member of the public noticed via drone-footage that a Kranji woodland had been cleared.^136^ In such cases, cancelling the development will not remedy the damage done, and there is little consideration of nature restoration solutions. In such cases, attempts to hold the government accountable has resulted in assurances that the government will ‘learn’ from this mistake.^137^ The focus is thus on the future, and what can be improved in the regime, not the particular decision itself.

Mechanisms for legal accountability thus seem more appropriate to properly interrogate why decisions were wrongfully made. The discussion of UK litigation in section 3 above clearly demonstrates how the courts can play an important role in holding decision makers to standards required by the law, and may complement avenues of political accountability as means of being heard when public opinion seems to have little influence over final decisions. The former has been affirmed in Singapore as well, where Chief Justice Chan (as he then was) suggested extrajudicially that ‘the courts play a supporting role by articulating clear rules and principles by which the Government may abide by and conform to the rule of law’.^138^ However, as mentioned earlier, the relatively strict standing requirements, limited political freedoms and generally nascent judicial culture frustrates the ability of the Singapore courts to do so in respect of the planning regime. On top of these factors, another important feature of the legal culture that creates a rift between the ways UK and Singaporean courts can hold planning authorities accountable is the nature of planning legislation.

As outlined earlier, the normative logic of planning legislation can often be clear. The National Policy Statement framework under the Planning Act 2008 was meant to expedite NSIP decision-making processes. Hence, a purposive reading of the statute meant that the other government policies that were due for consideration under the framework had to be limited.^139^ The wider legislative mechanisms within the planning regime also frequently regard the discretion of planning authorities—whether local or executive—to exercise their discretion in a well-reasoned way to weigh between a multitude of different policies, assessments and considerations. Although some might mock such a legal framework as enabling ‘democracy by lawsuit’,^140^ the reality is that these frameworks have developed in tandem with a political culture of deliberative democracy amidst an increasingly neoliberal UK.^141^ Such approaches mutually reinforce the relationship between regulators and the regulated: the quality of public participation is expected to inform the quality and acceptance of public decisions.^142^ Hence, the courts also often refer to the democratic nature of participation, the responsibility and autonomy of local authorities, and the transparency required in publicity requirements at various stages of decision making.^143^

The same sort of legal frameworks are hardly present in Singapore’s planning regime: recall that the Master Plan is the only statutory plan, and that objections must be raised within a two-week window,^144^ after which they may not even be addressed with a public inquiry. Unlike the UK planning regime, the Singaporean legislative frameworks do not provide nor imply the level of procedural rigour or transparency that would allow the public to scrutinise how planning decisions are made, and to thereby question the rules and principles by which the government should abide. Rather, the bare-bones legal framework seems to imply that the normative logic behind the legislation envisions that the executive and its administrative agencies should continue to wield a strong discretion over the entire planning process, as there are few frameworks for transparency and public consultation. The above-mentioned objections to Singaporean planning decisions illustrate this: groups like the Nature Society had to offer their own reasons for why the development should not be pursued rather than scrutinise the reasons provided by the government; alternatively, the public may object to a development by raising concerns over what they observe to be poor conduct in the process of construction.^145^ This is not the same as the way the claimants in *Heathrow* had observed gaps in the government’s reasons under the Airport National Policy Statement. There are thus far fewer procedural obligations and normative standards within the Singaporean legislative framework by which decision makers can be held accountable for the ways their decisions are made.^146^

As a result, the dominant avenue by which decision makers are held accountable in the Singaporean planning regime is through informal channels. They are also held accountable primarily for their decisions, and not so much for the way they arrive at them. This reflects Sudo’s observation that ‘once elected, the task of governing should be left to the governing elite who will exercise independent judgment on what is in the long-term interest of the people and act on that basis’.^147^ Singapore’s planning success is enabled not merely by faith in the government’s metropolitan dreams, but by a planning regime that is aligned with and contributes to its non-litigious culture.

This is clearly not the same for the UK, where the planning regime features multiple avenues to hold decision makers accountable for how they make their decisions. Any proposed reform of either planning regime must therefore build upon the existing regulatory approaches and thinking. In the UK’s context, planning reform cannot transform its legal culture entirely—legal and political history cannot simply be re-legislated. Instead, reform should be attentive to the ways in which process-focused decision making can be expedited through consolidation, as it is clear that the legal culture is one where public participation and judicial review play important roles in holding decision makers accountable. Such accountability avenues require expertise and funding in order to realise their objectives. Yet, previous waves of UK planning reform have not provided local government with consistent and adequate financial resources to realise planning objectives.^148^ This is not to say that there is greater mistrust in the government in the UK, but it does reflect the wider basis for which decisions and law are legitimate.

### B. Legitimacy of Planning Authorities and Law

Authorities and the laws they make must purport to have some legitimacy in order to make the claim that those laws should be followed.^149^ However, legitimacy is a hotly contested concept. This article does not purport to offer an authoritative or exhaustive account of legitimacy, but merely suggests that the UK and Singapore legal cultures have different ways of legitimating their authorities and laws. To do so, it is helpful to outline three ways in which legitimacy is observed.^150^ First, legitimacy may be sourced from the ‘inputs’ of a regime. Scholars have generally regarded this to refer to democratically elected law makers, but a similar argument may be made of technocratic law makers.^151^ Citizens must believe that law makers are entitled to make laws, often because they had chosen these law makers themselves, or because the law makers have particular expertise to do so. Second, ‘through-put’ legitimacy refers to ‘a focus on how disruption is managed as fairly as possible, on how disputes are resolved, and on how consensus is built’.^152^ Planning law and decisions may thus also be legitimated by regimes that explain how the law and its outcomes are rationally and fairly arrived at, and by providing avenues to hold decision makers accountable. Finally, ‘output’ or performance legitimacy refers to how authorities and law may be legitimated by ‘effective implementation of policies that address and respond to the public interest’.^153^

Immediately, it is clear that both Singapore and the UK, having democratic systems of governance, may justify that their planning regimes are legitimated through ‘input’ legitimacy. The matter may be put more strongly in Singapore’s case, where the close synergy between its administrative bureaucracy and stable political leadership means that a vote for the government is also a vote for the administrative authorities. In contrast, the different sources of authority in the UK planning regime, from changing governments, local authorities who create local development plans, direct channels for the public offer feedback and the role of EU regulation, seem to suggest that the democratic legitimacy of the planning regime is more complex. For instance, although the Airport National Policy Statement was prepared by the government, it still received multiple objections across political and legal channels.

However, this does not suggest that planning decisions are less legitimate in the UK; rather, the process of critiquing such decisions is part of the legitimating process—ie through-put legitimacy. The final outcomes of planning policies and decisions are legitimate because they have passed through the various checks and balances put in place by the planning regime.^154^ Legitimacy for English planning decisions thus takes account of factors beyond the diverse sources of input legitimacy or evaluations of the outcome itself. In contrast, the Singapore planning regime, in priding itself on its ability to have a comprehensive metropolitan vision, is clearly also legitimised through the implementation of high-quality planning decisions that accurately anticipate and address the public’s needs. But sources of ‘through-put’ legitimacy are minimal in the Singaporean planning regime as decisions are often not made in a transparent manner, public consultations are disparate and it is more difficult overall to challenge planning policies and decisions. Instead, the combination of a technocratic set of experts—comprising both administrators and elected officials—and a track record of achieving Singapore’s metropolitan dreams has legitimised the planning regime by framing the regime as an extension of a ‘pragmatic’ government.^155^

The way the planning regimes are legitimised poses questions as to existing and future deficits in their legitimacy. In Singapore’s case, the relative lack of avenues to hold authorities accountable has clearly contributed to a palpable deficit in legitimacy. After all, if the government-led planning decisions were legitimate by virtue of the government’s consistently high electoral vote share or the high quality of its plans, then why has there been a recent uptick in the number of public consultations held for planning policies and projects?^156^ Although one may argue this is because public consultation can result in better decisions, or it would quell subsequent objections to planning decisions, the fact that it is unclear how public feedback is addressed, let alone implemented, suggests that the reasons for this policy shift are not entirely instrumental in nature. It is thus suggested that the reason for the increased frequency of public consultations is to introduce greater legitimacy to the planning regime.

While Singapore seeks to address its deficits in legitimacy, the UK seems to be risking its existing claim to a legitimate planning regime through its proposed planning reforms. The prospect of output legitimacy through the delivery of much-needed infrastructure is not a unique proposition of abundance theorists or the new UK executive. Rather, since the 1990s, various UK administrations have engaged in planning reform to address persistent gaps in housing^157^—gaps that continue to exist today, warranting the proposed reform of the Planning and Infrastructure Bill. Scepticism over executive-led planning had given way to the deliberative democratic procedures under the Localism Act 2011. Yet, later governments would blame those reforms for holding the UK’s metropolitan dreams back.

History may repeat itself. If the focus was solely on development outcomes led by a strong executive works, then the infrastructure crisis faced by the UK would have been solved in earlier editions of planning reform. The lack of attention given to the relationship between such outcomes and the factors relevant to input and through-put legitimacy reveals both gaps and opportunities. Local-led planning initiatives were satisfactory when local government and communities were adequately funded: multi-stakeholder decision making can only be effective when parties have the relevant legal and financial means to advocate for themselves throughout the planning process.^158^ When these processes are compromised, the courts play an important role in ensuring decision making remains fair for all stakeholders involved.^159^ The roles of courts and stakeholders in the planning process should not be reduced to ‘blockers’ and ‘serial objectors’.^160^ Reform that primarily targets the expediting of planning permission fails to pay heed to the role that various stakeholders play in investigating environmental impacts, hearing proposed schemes and overseeing the construction of a proposed development. Planning is a process, not merely an outcome. Processes take time and financial resources, not just a strong vision.

Input, through-put and output legitimacy are thus deeply interrelated with one another in the planning context. The ability for the legal regime to achieve its planning outcomes is reliant on how input and through-put factors map onto socio-political contexts. Where the planning regime is subject to the vagaries of politics, democratic mandates and political promises of development ought to be closely scrutinised by the people and the courts. Such processes are important for legal regimes and their outcomes to maintain legitimacy. Legitimacy is important for legal regimes not just because it justifies an authority’s claim to its law-making powers, but the political legitimacy of laws and decisions can be a proxy for the acceptance of those decisions.^161^ Deregulatory reform may mean fewer legal procedures in one sense, but it does not guarantee legal and political disagreement in other forums. Stakeholders will continue to find other means to hold legislators and administrative agencies accountable for their decisions because such reform is unlikely to alter the role and rule of law.

### C. Role and Rule of Law

A discussion on the role and rule of law for Singapore and the UK is a Herculean task—particularly because the rule of law is an unsettled concept. This subsection thus only considers the application of ideas on the rule of law to the planning regime to thereby distill how the roles and rule of law explain why changes to the planning regime, in order to achieve metropolitan dreams, can be slow.

The rule of law generally refers to how all are equal before the law rather than subject to the exercise of arbitrary power.^162^ In the context of planning regimes, this brings to mind the ways in which decision makers are held accountable, and the laws that bestow decision makers with their authority and powers. Given that dominant conceptions of accountability and the ways planning legislation is crafted in the UK and Singapore are vastly different, this also indicates that the rule of law takes different forms in both states. At the outset it can be noted that for Singapore the frequency in which political accountability is relied upon and the ways in which the planning regime is legitimised demonstrate how the rule of law is appreciated in utilitarian and functional terms^163^—in the words of Singapore’s Chief Justice, law is merely a facilitative tool to achieve Singapore’s ‘development narrative’ and ‘emergence as a modern economic miracle’.^164^ Planning legislation thus provides a skeletal framework to empower expert administrative agencies to work with the government towards their common planning goals.

In contrast, in the UK planning regime, the rule of law takes on a more substantive form by ensuring normative ideals like those of democracy and fairness are upheld.^165^ The purpose of planning legislation is to empower various stakeholders (local authorities, the public) to participate in the decision-making process, and to scrutinise the executive and its administration. It creates a complex system of accountability by which both the ways in which decisions are made and the decisions themselves are held to particular standards.

Why is this distinction important? The reasons why the frameworks of the planning regime, the ways the public continue to hold authorities accountable and the sources of the regime’s legitimacy are not liable to dramatic change relate back to the rule of law. The rule of law, as a feature of a particular legal culture, generally endures amidst legal and policy reform.^166^ Although the exact shape of the planning legal regime may shift, the endurance of the rule of law speaks to the quality of governance or risks of ‘state failure’.^167^ Attempts to expedite particular NSIP planning procedures will not eliminate the existing dissent against a particular development due to suspicions over executive decision making; it may merely change the ways it is challenged. For instance, in the aftermath of the UK Supreme Court’s decision in the *Heathrow* litigation, labour and environmental groups are still speaking out against the Chancellor’s desire to resume plans to expand the airport.^168^

The comparison with Singapore’s planning regime and constitutional context also points towards the difficulty of moving between substantive and formal conceptions of the rule of law. Despite enthusiasm from the Singaporean courts to uphold principles of good administration, the occasions on which they can do so have been few and far between. Even where the judicial culture has evolved to include new heads of review, like that of legitimate expectations, the success of those heads of claim has yet to be seen for the planning regime.^169^ Revisiting *Chiu Teng*, the court held that a prior representation via the administrative authority’s circular on how a ‘differential premium’ was to be calculated did not amount to a legitimate expectation, and that the authority was entitled to calculate the premium on an entirely different basis that was never disclosed to the claimant despite his failed attempts to contact the authority. In reaching this conclusion, the court decided that the authority’s circular had disclaimed in its ‘Terms of Use’ that it did not amount to ‘any representations or warranties whatsoever’, and that the applicant, who was a property developer seeking to redevelop land, would have known better.^170^ The applicant’s failure in *Chiu Teng* recalls this article’s observation that Singaporean planning legislation does not introduce procedural frameworks that would guarantee citizens a right to information or that would hold the authority to particular standards. Citizens will rarely have ‘legitimate expectations’ when the administrative authorities can easily argue that there was never any legislation that made a promise, and where authorities have the discretion and flexibility to renege on policies. There are simply too few normative hooks and procedural frameworks by which principles of good administration may be gleaned or participation of the public enshrined. While the Singapore courts may think of themselves as ‘the sharp edge that keeps government action within the rule of law’, the reality for planning decisions is that there simply is not much law to keep government action in line with.^171^ This disjunct means that the existing constitutional and legal culture endures.

‘Singapore on Thames’ may thus refer to a transformation of the UK’s cityscape to that of Singapore’s, but it cannot include a similar change in its constitutional landscape. Because the role of law is so different in the UK and Singapore, so too is the conception of the rule of law. Changes to the planning regime reflect a shift in law makers’ understanding of the role of law, but it does not necessarily mean the rule of law will or must adapt to such a shift. Not only does the rule of law withstand the tests of time, it also meets out its own tests against public governance. Both administrative law and the politics that underscore it must answer to its scrutiny in unison.^172^ As history has shown, disharmony in the planning regime can crush England’s metropolitan dreams.^173^

## 5. Conclusion

In the King’s Speech of July 2024, King Charles III proclaimed that ‘securing economic growth will be a fundamental mission … Ministers will get Britain building, including through planning reform, as they seek to accelerate the delivery of high quality infrastructure and housing’.^174^ The spirit of this reform lay in deregulation by reformulating reporting requirements, reducing avenues for judicial review and providing the executive with greater control of the planning agenda. When taken to the extreme, each of these techniques resemble Singapore’s planning regime, where reporting is generally left to the discretion of the executive, there is minimal judicial review and intervention, and the executive has a firm grasp on the short- and long-term planning policies for the nation.

When putting the UK and Singaporean planning regimes side by side, it is possible to chart a linear path towards a ‘Singapore on Thames’ by suggesting that greater deregulation and executive control can help the UK move towards its metropolitan dreams like Singapore has. But such deregulatory agendas fail to account for the multitude of factors that have enabled the Singaporean planning regime to function the way it has. The legal frameworks of the planning regime, close connection between administrative agencies and the ruling government, a political culture that is relatively less hostile to government decisions and a legal culture that ensures the law facilitates the government’s decisions rather than frustrates them are all reasons why the planning regime can deliver on its achievements. The ability of a planning regime to achieve development goals is thus a complicated matter. Indeed, in the words of Singapore’s ‘Father of City Planning’, planning is not just about ‘[drawing] lines and [putting] colours on paper’; it is about providing ‘sufficient explanation or justification’.^175^

However, the ways in which explanations and justifications for planning decisions are provided can vary greatly across legal regimes. Singapore’s planning decisions may be well reasoned, but there are few legal structures that ensure those reasons are well publicised, nor standards by which one can measure how well they are reasoned. In contrast, such structures do exist and are often relied upon in the UK’s planning regime. Law provides avenues to scrutinise the decision-making process and outcomes amidst changing political agendas and legal reform. Attempts to introduce executive-led acceleration of infrastructure delivery in the UK risks underestimating the deeply rooted systems of accountability, legitimacy and rule of law—reducing planning to ‘drawing lines and putting colours on paper’.

The differences in the legal cultures of both countries demonstrates how trust in government, and hence its planning decisions, takes different forms. Trust may be gained from a series of early successes, like the story of Singapore’s historic success in urban planning, thereby legitimising a bare-bones legal planning regime. Trust may be placed in the judicial system and rule of law rather than in executive and administrative discretion because of a ‘thick’ rule of law that prioritises normative values like democracy and transparency. Trust is earned, but it may also be lost. That is why both the UK and Singapore’s planning regimes are not static. Both regimes are flirting with change by introducing measures that invite the public to trust the government, either by engaging with the public directly or by giving the executive more control over the planning regime.

The UK’s reforms seem to require a certain level of trust in the government for planning processes and decisions to truly be expedited, but this trust may not exist as the existing planning regime already sprawls beyond the executive. Like the Thames, input from many different channels flows into a single planning decision so that it is difficult to say only one source is responsible for the whole regime. Merely widening its source may not guarantee such a regime’s success. Greater support, through both legal and non-legal resources, across all these channels is necessary for the entire system to thrive. A planning regime like Singapore’s, with its distinct legal culture and relative political inactivity, may not fit easily in the dynamic environment of the Thames. Consequently, rather than a slogan for its economic and metropolitan dreams, ‘Singapore on Thames’ might just be an oxymoron of the times.

## Footnotes

1 Jill Rutter, ‘Singapore on Thames Is Dead, Long Live Singapore on Thames’ (*UK in a Changing Europe*, 27 October 2020) <https://ukandeu.ac.uk/singapore-on-thames-is-dead-long-live-singapore-on-thames/>.

2 Jeevan Vasagar, ‘Singapore-on-Thames? This Is No Vision for Post-Brexit Britain’ *The Guardian* (24 November 2017) <www.theguardian.com/commentisfree/2017/nov/24/singapore-on-thames-post-brexit-britain-wealthy-city-state>.

3 Matt Withers, ‘Jeremy Hunt Hails SINGAPORE as the Model for a Post-Brexit Britain’ *The New European* (30 December 2018) <www.theneweuropean.co.uk/brexit-news-jeremy-hunt-hails-singapore-as-the-model-for-a-post-38102/>.

4 John Christensen, ‘Singapore-on-the-Thames: Slogan or Strategy’ in A Nesvetailova and others (eds), *A Singapore on the Thames? Post-Brexit Deregulation in the UK* (CityPerc 2017) 28.

5 Thum Ping Tjin, ‘Singapore: A Model for Post-Brexit Britain?’ (*New Naratif*, 25 November 2019) <https://newnaratif.com/singapore-a-model-for-post-brexit-britain/>.

6 Woo Jun Jie, ‘Singapore’s Transformation into a Global Financial Hub’ (Lee Kuan Yew School of Public Policy 2017) 11 <https://lkyspp.nus.edu.sg/docs/default-source/case-studies/entry-1516-singapores_transformation_into_a_global_financial_hub.pdf?sfvrsn=a8c9960b_2>.

7 See generally Govinda Timilsina and others, ‘How Much Does Physical Infrastructure Contribute to Economic Growth?’ (World Bank 2021).

8 Prime Minister’s Office, ‘PM: Build, Build, Build’ (30 June 2020) <www.gov.uk/government/news/pm-build-build-build>.

9 Ross Hawkins, ‘Boris Johnson Defends “Long Overdue” Planning Overhaul in England’ (*BBC News*, 7 August 2020) <www.bbc.com/news/uk-53669432>.

10 As of writing, the Bill is being read in the House of Commons. Planning and Infrastructure Bill 2025, Bill 196 2024–25 <https://publications.parliament.uk/pa/bills/cbill/59-01/0196/240196.pdf>.

11 Joël Reland, ‘The strange rebirth of Singapore-on-Thames’ *UK in a Changing Europe* (14 January 2025) <https://ukandeu.ac.uk/the-strange-rebirth-of-singapore-on-thames/>.

12 Joseph Chun and Lye Lin Heng, *Environmental Law in Singapore* (SAL Publishing 2019) 308.

13 Neil Komesar, ‘In Search of a General Approach to Legal Analysis: A Comparative Institutional Alternative’ (1981) 79(7) Mich L Rev 1350.

14 Gillian Koh, ‘Bureaucratic Rationality in an Evolving Developmental State: Challenges to Governance in Singapore’ (1997) 5(2) Asian Journal of Political Science 114, 120.

15 Jessica Ann Diehl and Isabelle Chan, ‘Is It Just Apathy? Using the Theory of Planned Behaviour to Understand Young Adults’ (18 to 35 years old) response to government efforts to increase planning participation in Singapore’ (2021) 1 Urban Governance 89, 95.

16 eg John Sturzaker and Michael Gordon, ‘Democratic Tensions in Decentralised Planning—Rhetoric, Legislation and Reality in England’ (2017) 35(7) Environment and Planning C: Politics and Space 1324.

17 David Nelken, ‘Using the Concept of Legal Culture’ (2009) 29 Australian Journal of Legal Philosophy 1.

18 Ralf Michaels, ‘The Functional Method of Comparative Law’ in Mathias Reimann and Reinhard Zimmermann (eds), *The Oxford Handbook of Comparative Law* (OUP 2012).

19 eg *Kennedy v Charity Commission* [2014] UKSC 20 [1].

20 As part of the ‘green-light’ theory of judicial review. Chan Sek Keong CJ, ‘Judicial Review—From Angst to Empathy’ (2010) 22 SAcLJ 469, 480.

21 While the conclusions of this article may resemble similar conclusions in the legal transplant scholarship, this article does not draw on nor frame the matter of the UK’s reforms as legal transplants. eg Pierre Legrand, ‘The Impossibility of “Legal Transplants”’ (1997) 4 MJ 111.

22 Maria Lee, ‘The Importance of Taking English Planning Law Scholarship Seriously’ in Maria Lee and Carolyn Abbot (eds), *Taking English Planning Law Scholarship Seriously* (UCL Press 2022) 3.

23 For instance, scholarship on Singapore’s planning regime tends to focus on the politics or urban planning that enabled its development. eg Orlando Woods and Lily Kong, ‘Mobile Cities, Modelling Policies: Importing/Exporting the Singapore “Model” of Development’ in John Short (ed), *A Research Agenda for Cities* (Edward Elgar 2017) 206.

24 Sean Michael, ‘Trump Directive Aims to Speed Up Deregulation by Nixing Public Input’ (*Government Executive*, 10 April 2025) <www.govexec.com/management/2025/04/trump-directive-aims-speed-deregulation-nixing-public-input/404474/>.

25 eg Ezra Klein and Derek Thompson, *Abundance* (Avid Reader Press 2025).

26 Anders Esmark, ‘Maybe It Is Time to Rediscover Technocracy? An Old Framework for a New Analysis of Administrative Reforms in the Governance Era’ (2017) 27(3) Journal of Public Administration Research and Theory 501.

27 eg Michael Barr, ‘Beyond Technocracy: The Culture of Elite Governance in Lee Hsien Loong’s Singapore’ (2006) 30(1) Asian Studies Review 1.

28 Liz Fisher, ‘In Praise of Legal Parochialism: On Why I Read the Decisions of Specialist Courts’ in T Mann (ed), *Enduring Courts in Changing Times* (Australian Academy of Law 2024).

29 Cary Coglianese and others, ‘The Deregulation Deception’ (Penn Program on Regulation Research Report, 1 November 2020) <https://pennreg.org/wp-content/uploads/2022/11/CoglianeseSarinShapiroTrumpDeregulationReport110120.pdf>.

30 I am grateful to Liz Fisher for sharing her ideas on parochialism in administrative law scholarship. See generally Liz Fisher, ‘Law’s Importance Does Not Come from It Being Wrapped in Plastic: On Future Proofing Environmental Law’ in K Huhta and others (eds), *Future-Proofing Law in a Time of Environmental Emergency* (Edward Elgar 2025); Liz Fisher, ‘The Open Road? Navigating Public Administration and the Failed Promise of Administrative Law’ in Liz Fisher, Jeff King and Alison Young (eds), *The Foundations and Future of Public Law: Essays in Honour of Paul Craig* (OUP 2020) 211.

31 eg ‘If Britain Became “Singapore-on-Thames”: The British Economy if the Country Crashes Out of the European Union’ *The Economist* (13 July 2017) <www-economist-com.libproxy1.nus.edu.sg/the-world-if/2017/07/13/the-british-economy-if-the-country-crashes-out-of-the-european-union>.

32 Lucy Burton, ‘Cheer Up! London Could Be the New “Super Singapore”’ *Financial News* (10 September 2016) <www.fnlondon.com/articles/brexit-john-redwood-howard-flight-shearman-sterling-20160915>.

33 John Springford, ‘The EU Should Not Fret about Singapore-on-Thames’ (Centre for European Reform, 28 September 2018) <www.cer.eu/publications/archive/bulletin-article/2018/eu-should-not-fret-about-singapore-thames>.

34 See generally Claudia Goldin and Gary Libecap, *The Regulated Economy: A Historical Approach to Political Economy* (Chicago Press 1994).

35 See generally Karine Tournier-Sol and Chris Gifford, *The UK Challenge to Europeanization* (Springer 2015).

36 Brian Burgoon, ‘Inequality and Anti-globalization Backlash by Political Parties’ (2013) 14(3) European Union Politics 408, 410.

37 Tom Nichols, *The Death of Expertise: The Campaign against Established Knowledge and Why It Matters* (2nd edn, OUP 2024) 260; Donald Moynihan, ‘Delegitimization, Deconstruction and Control: Undermining the Administrative State’ (2022) 699 Annals Am Acad Pol & Soc Sci 36, 49.

38 eg Frank Thompson and others, *Trump, the Administrative Presidency, and Federalism* (Brookings Institution 2020).

39 Denis Hew, ‘Singapore as a Regional Financial Centre’ in D Vandenbrink and D Hew (eds), *Capital Markets in Asia: Changing Roles for Economic Development* (ISEAS 2005).

40 Linus Pardoe, ‘Seven Lessons from Singapore: What Britain Can Learn’ (Policy Commons, 2021) <https://policycommons.net/artifacts/1728404/seven-lessons-from-singapore/2460049/>.

41 eg Jiangyu Wang, ‘The Rise of Singapore as International Financial Centre Political Will, Industrial Policy, and Rule of Law’ in Jiaxiang Hu and others (eds), *Finance, Rule of Law and Development in Asia* (Brill 2016).

42 Maria Lee (n 22).

43 Urban Redevelopment Authority, ‘Master Plan 2019: Themes’ (2025) <www.ura.gov.sg/Corporate/Planning/Master-Plan/Master-Plan-2019/Themes>.

44 Ministry of Housing, Communities & Local Government, ‘Guide to the Planning and Infrastructure Bill’ (2025) <www.gov.uk/government/publications/the-planning-and-infrastructure-bill/guide-to-the-planning-and-infrastructure-bill’.

45 Jessica Elgot, ‘Heathrow Third Runway: A Saga of Promises, Protest and U-Turns’ *The Guardian* (29 January 2025) <www.theguardian.com/uk-news/2025/jan/29/heathrow-third-runway-a-saga-of-promises-protest-and-u-turns>.

46 Lilit Marcus, ‘“Mega yet Cozy”: Singapore’s Changi Airport Breaks Ground on New Terminal’ (*CNN*, 16 May 2025) <https://edition.cnn.com/2025/05/15/travel/singapore-changi-airport-new-terminal-breaks-ground>.

47 Eric Thompson, ‘Singaporean Exceptionalism and Its Implications for ASEAN Regionalism’ (2002) 28(2) Contemporary Southeast Asia 183.

48 Klein and Thompson (n 25) 25–8, 81–4.

49 While NSIPs specifically refer to large-scale projects in the fields of ‘energy, transport, water, wastewater, waste’ (per s 14(6) of the Planning Act 2008 (UK)) so that a dedicated procedure for planning and obtaining development consent is provided for. No such equivalent regime exists in Singapore, but the concept of a ‘significant’ project is present across political statements on the planning regime.

50 Susan Fanstein and James DeFilippis, *Readings in Planning Theory* (Blackwell Publishing 2003) 12.

51 Patrick McAuslan, *The Ideologies of Planning Law* (Pergamon Press 1980) 265.

52 Liz Fisher, ‘Environmental Law as “Hot” Law’ (2013) 25(3) JEL 347.

53 Directive 2011/92/EU.

54 Directive 200142/EU.

55 Designing Buildings, ‘Local Development Framework LDF’ (2022) <www.designingbuildings.co.uk/wiki/Local_development_framework_LDF>.

56 ibid. Planning and Compulsory Purchase Act 2004, ss 17–19 (UK).

57 Ministry of Housing, Communities & Local Government, ‘National Planning Policy Framework’ (December 2024) <https://assets.publishing.service.gov.uk/media/67aafe8f3b41f783cca46251/NPPF_December_2024.pdf>.

58
*Save Britain’s Heritage v Liverpool CC* [2016] EWCA Civ 806 [26].

59
*Friends of the Earth v Heathrow Airport* [2020] UKSC 52 [115], referring to Planning Act 2008, s 10 (UK).

60 Planning Act 2008, s 104(2) (UK).

61 HM Government, *Planning for a Sustainable Future* (White Paper, Cm 7120, 2007) [3.12] <https://assets.publishing.service.gov.uk/media/5a7c971d40f0b6629523a80e/7120.pdf>.

62 Levelling Up and Regeneration Act 2023, s 94 (UK).

63 ibid s 93.

64 ibid s 94(3).

65 Tim Marshall and Richard Cowell, ‘Infrastructure, Planning, and the Command of Time’ (2016) 34 Environment and Planning C: Politics and Space 1843.

66 In relation to National Policy Statements, see Planning Act 2008, ss 7–8 and 13 (UK); for local development frameworks, see Planning and Compulsory Purchase Act 2004, s 113(3) (UK).

67 Lucy Natarajan and others, ‘Participatory Planning and Major Infrastructure: Experiences in REI NSIP Regulation’ (2019) 90 Town Planning Review 117, 120.

68 Environmental Assessment of Plans and Programmes Regulations 2004, s 5(1) (UK);

69 Town and Country Planning (Environmental Impact Assessment) Regulations 2017, ss 8 and 18–20.

70
*Tesco Stores Ltd v Dundee City Council* [2012] UKSC 13.

71 Town County and Planning Act 1990, s 78 (UK).

72 Lord Frederick Ponsonby, ‘Infrastructure Planning and Judicial Review Reform’ (UK Parliament, 23 January 2025) Statement UIN HLWS382.

73
*Plan B Earth v Secretary of State for Transport* [2020] EWCA Civ 214 [283].

74
*Friends of the Earth* (n 59) [106].

75 Antonia Layard, ‘Planning and Environment at the Crossroads’ (2002) 14 JEL 401.

76
*Friends of the Earth* (n 59) [20]–[32].

77 Tim Marshall, ‘Infrastructure Futures and Spatial Planning: Lessons from France, the Netherlands, Spain and the UK’ (2014) 89 Progress in Planning 1, 23.

78 Klein and Thompson (n 25) 76–9.

79
*South Bucks District Council v Porter* [2003] UKHL 26 [10].

80 These themes are explored in greater depth in Section 4. Liz Fisher, Bettina Lange and Eloise Scotford, *Environmental Law: Text, Cases, Materials* (2nd edn, OUP 2019) 651.

81 John Dryzek, *Deliberative Democracy and Beyond: Liberals, Critics, Contestations* (OUP 2002).

82 eg *R (Mott) v Environment Agency* [2016] 1 WLR 4338 [181].

83 See ‘green-light theories’ of administrative law, Carol Harlow and Richard Rawlings, ‘Red and Green Light Theories’ in Carol Harlow and Richard Rawlings (eds), *Law and Administration* (CUP 2012) 31.

84 It is implicit in the idea that a legal environment should be calculable, not just predictable. Jeremy Waldron, ‘Are Sovereigns Entitled to the Benefit of the International Rule of Law?’ (2011) 22(2) EJIL 315, 324.

85 Planning Act 1998, ss 6–8 (Singapore).

86 See eg ‘2019 Master Plan: Urban Redevelopment Authority, Master Plan’ (14 February 2025) <https://eservice.ura.gov.sg/maps/?service=MP>.

87 Urban Redevelopment Authority, ‘Space For Our Dreams: Long-Term Plan Review 2021’ (2021) <www.ura.gov.sg/-/media/Corporate/Planning/LTPR21/LTPR_publication.pdf>.

88 Chun and Lye (n 12) 292.

89 Urban Redevelopment Authority (n 87) 4; Housing Development Board, ‘Designing for Life Roadmap’ (2025) <www.hdb.gov.sg/about-us/hdbs-refreshed-roadmap-designing-for-life>.

90 Planning Act 1998, s 14 (Singapore).

91 See generally A Haila, *Urban Land Rent: Singapore as a Property State* (Wiley 2015).

92 Planning Act 1998, ss 13–14 (Singapore).

93 Urban Redevelopment Authority, ‘Submission Requirements’ (2025) <www.ura.gov.sg/Corporate/Guidelines/Development-Control/Planning-Permission/using-CORENET-eSS/Submission-requirements>. See also eg Building Construction Authority, ‘BP Consultation with Technical Departments’ (2005) <www1.bca.gov.sg/docs/default-source/docs-corp-regulatory/building-control/rbca00006.pdf>.

94 Master Plan Rules 1999, s 4(a) (Singapore).

95 ibid s 6(1).

96 ibid s 6(3).

97 Chun and Lye (n 12) 292.

98 Urban Redevelopment Authority, ‘Long-Term Plan Review: Public Engagement Journey’ (2025) <www.ura.gov.sg/Corporate/Planning/Long-Term-Plan-Review/Public-Engagement-Journey>.

99 Desmond Lee, *Objective Criteria and Standards Used by NParks in Deciding Necessity for Environmental Impact Assessment*, Singapore Parliamentary Debates vol 95 (2 August 2023).

100 Public Order Act 2009, ss 5–19 (Singapore).

101 Jolene Lin, ‘The Judicialization of Governance: The Case of Singapore’ in Tom Ginsburg and Albert Chen (eds), *Administrative Law and Governance in Asia: Comparative Perspectives*, 350 (2009) 348. cf Chan (n 20). The extent to which the state is held accountable for its planning decisions before the courts is discussed in s 4.

102
*Tan Eng Hong v Attorney-General* [2012] 4 SLR 476 [69]–[80].

103
*Jeyaretnam Kenneth Andrew v Attorney-General* [2014] 1 SLR 345 [51] and [62].

104 Benjamin Ong, ‘Standing Up for Your Rights: A Review of the Law of Standing in Judicial Review in Singapore’ (2019) Sing JLS 319.

105 Planning Act 1998, s 21(4) (Singapore).

106
*Chiu Teng @ Kallang Pte Ltd v Singapore Land Authority* [2014] 1 SLR [63]–[64].

107 ibid [67]–[69].

108 eg Joan Henderson, ‘Planning for Success: Singapore, The Model City-State’ (2012) 65(2) Journal of International Affairs 69.

109 Tan Jake Hooi, ‘Metropolitan Planning in Singapore’ (1996) 4(4) Australian Planning Institute Journal 111, 118.

110 See generally *Parliamentary Elections* <https://sg-elections.com/>.

111 Lin (n 101).

112 Woo Jun Jie, ‘Singapore: Administrative City-State’ in Andreas Ladner and Ffritz Sager (eds), *Handbook on the Politics of Public Administration* (Edward Elgar 2021) 353–7.

113 ibid 352.

114 No clearer legal or policy framework for environmental impact assessment has emerged as of the time of writing. A Tan, ‘Environmental Impact Assessments in S'pore to Be Further Strengthened: Desmond Lee’ *The Straits Times* (2021) <www.straitstimes.com/singapore/environment/environmental-impact-assessments-to-be-further-strengthened-desmond-lee>.

115 Levelling Up and Regeneration Act 2023, s 153 (UK).

116 Rhiannon Ogden-Jones, ‘Policies, Places and Practices: Why Environmental Law Scholars Should Care About the Levelling-Up and Regeneration Act 2023’ (2024) 36 JEL 445, 450.

117 ibid 451.

118 Town and Country Planning (Environmental Impact Assessment) Regulations 2017, s 3.

119 Levelling Up and Regeneration Act 2023, s 156.

120 Maia Perraudeau, ‘Back to the Future: Brexit, EIA and the Challenge of Environmental Judicial Review’ (2019) 21(1) Env L Rev 6.

121 John Benson, ‘What Is the Alternative? Impact Assessment Tools and Sustainable Planning’ (2003) 21 Impact Assessment and Project Appraisal 261.

122 Ministry of Housing, Communities and Local Government, ‘Pro-growth Package Unshackling Britain to Get Building’ (13 October 2025) < www.gov.uk/government/news/pro-growth-package-unshackling-britain-to-get-building>.

123 Planning and Infrastructure Bill 2025, ss 4 and 47, 12D.

124 Ministry of Housing, Communities & Local Government, ‘Guide to the Planning and Infrastructure Bill’ (24 April 2025) part 2.

125 Planning and Infrastructure Bill 2025, s 8.

126 Joanne Hawkins, ‘Nationally Significant Infrastructure Projects and the Planning and Infrastructure Bill: A Move in the Wrong Direction’ (2025) 27(3) Env L Rev 224.

127 ibid 228.

128 Klein and Thompson (n 25) 84.

129 Tim Marshall, *The Politics and Ideology of Planning* (OUP 2020) ch 6.

130 Klein and Thompson (n 25) 76–9.

131 Nicholas Bagley, ‘The Procedure Fetish’ (2019) 118 Mich L Rev 345, 350; Maria Lee (n 22) 5–6.

132 Anne Davies, *Accountability: A Public Law Analysis of Government in Contract* (OUP 2001) 75.

133 See generally Derick Brinkerhoff, ‘Taking Account of Accountability: A Conceptual Overview and Strategic Options’ (USAID 2001) 4.

134 Wee Yeow Chin and Richard Hale, ‘The Nature Society (Singapore) and the Struggle to Conserve Singapore’s Nature Areas’ (2008) 1 Nature in Singapore 41, 42.

135 eg Woo Junjie, ‘Commentary: Saving Dover Forest and the Plight of the Singapore Urban Planner’ *The Straits Times* (30 January 2021) <www.channelnewsasia.com/commentary/dover-forest-singapore-conservation-nature-park-hdb-ura-1882941>.

136 Tan (n 109).

137 Desmond Lee (n 99).

138 Chan (n 20) 480.

139
*Friends of the Earth* (n 76).

140 Klein and Thompson (n 25) 76; Bagley (n 131) 351–7.

141 Marshall, ‘Infrastructure Futures and Spatial Planning’ (n 77).

142 Carolyn Abbott, ‘Losing the local? Public participation and legal expertise in planning law’ (2020) 40(2) Legal Studies 269.

143 Fisher, Lange and Scotford (n 80).

144 In practice, this is extended to four weeks.

145 See also the Nature Society’s challenge to build a road through Bukit Brown Cemetery. Nature Society, ‘Nature Society’s Position’ (*bukitbrown.com*, 23 March 2012) <https://bukitbrown.com/main/the-nature-society-singapore-on-bukit-brown/>.

146 Kenny Chng, ‘Conceptualising a Role for the Common Law in Environmental Protection in Singapore’ (2021) 16(2) ASJCL 1.

147 Sueo Sudo, ‘Regional Governance and East and Southeast Asia: Towards the Regulatory State?’ (2004) 4(2) Japanese Journal of Political Science 331, 341.

148 Colin Talbot, ‘The Hidden Crisis in English Local Government’ (The Constitution Society, 31 May 2024) <https://consoc.org.uk/hidden-crisis-in-english-local-government/>.

149 See generally Joseph Raz, *Authority of Law: Essays on Law and Morality* (OUP 1979).

150 Liz Fisher, ‘Challenges for the EU Climate Change Regime’ (2020) 21 German Law Journal 5.

151 Susan Rose-Ackerman, ‘Citizens and Technocrats: An Essay on Trust, Public Participation, and Government Legitimacy’ in Susan Rose-Ackerman, Peter L Lindseth and Blake Emerson (eds), *Comparative Administrative Law* (Edward Elgar 2017).

152 Fisher, ‘Challenges’ (n 150) 8.

153 Erik Martinez Kuhonta, ‘Performance Legitimacy and Coercion in the Authoritarian States of East and Southeast Asia’ in Anne Wolf (ed), *The Oxford Handbook of Authoritarian Politics* (OUP 2024) 1.

154 Philip Booth, ‘Planning and the Rule of Law’ (2016) 17(3) Planning Theory and Practice 344, 357.

155 Kenneth Paul Tan, ‘The Ideology of Pragmatism: Neo-liberal Globalisation and Political Authoritarianism in Singapore’ (2012) 42 Journal of Contemporary Asia 67.

156 Chun and Lye (n 12) 63–7; J Leeming, ‘Singapore’s Fight to Save Its Green Spaces from Development’ (*Nature*, 28 May 2025) <www.nature.com/articles/d41586-025-01578-y>.

157 Olivier Sykes and John Sturzaker, ‘Introduction’ in Olivier Sykes and John Sturzaker (eds), *Planning in a Failed State* (CUP 2025).

158 John Sturzaker and Olivier Sykes, ‘Localism: The Peccadillos of a Panacea’ in ibid.

159 Antonia Murillo, ‘Reforming the Judicial Review Process: Town and Country Planning 2013–2018’ (PhD thesis, University of Sussex 2025) <https://sussex.figshare.com/articles/thesis/Reforming_the_judicial_review_process_town_and_country_planning_2013_-_2018/29221400?file=55072676>.

160 HM Treasury, ‘Chancellor Takes On the Blockers to Get Britain Building’ (15 October 2025) <www.gov.uk/government/news/chancellor-takes-on-the-blockers-to-get-britain-building>.

161 While interrelated, political legitimacy and acceptance have different normative and operational implications that are outside the scope of this article. See R Betancourt and A Ponce, ‘Political Acceptance as an Alternative or Complement to Political Legitimacy: Concept, Measurement and Implications’ (Society for Institutional & Organizational Economics 2014) <https://extranet.sioe.org/uploads/isnie2014/betancourt_ponce.pdf>.

162 AV Dicey, *Lectures Introductory to the Study of The Law of the Constitution* (JWF Allison ed, OUP 2013) 119.

163 Li-Ann Thio, ‘Rule of Law within a Non-liberal “Communitarian” Democracy: The Singapore Experience’ in Randall Peerenboom (ed), *Asian Discourses of Rule of Law* (Routledge 2004) 183.

164 Sundaresh Menon CJ, ‘The Rule of Law: The Path to Exceptionalism’ (2016) 28 SAcLJ 413, 419.

165 Booth (n 154) 351–7.

166 For a similar idea, see Keith Ewing, ‘The Resilience of the Political Constitution’ (2013) 14 German Law Journal 2113.

167 United Nations Human Settlements Programme, ‘Leading Change: Delivering the New Urban Agenda through Urban and Territorial Planning’ (UN 2018) 79; Sykes and Sturzaker (n 157) 12.

168 Unite the Union, ‘Reeves Speech: Heathrow Expansion Welcome but Lack of Industrial Strategy Threatens Jobs, Skills and Communities’ (29 January 2025) <www.unitetheunion.org/news-events/news/2025/january/reeves-speech-heathrow-expansion-welcome-but-lack-of-industrial-strategy-threatens-jobs-skills-and-communities>; WWF, ‘WWF Statement on Why Airport Expansion Will Not Spark Growth’ (4 February 2025) <www.wwf.org.uk/press-release/wwf-reacts-airport-expansion-will-not-spark-growth>.

169
*Chiu Teng* (n 106) [107]–[111].

170 ibid [120]–[121].

171 Menon (n 164) 421–3.

172 Bagley (n 131) 358–68.

173 Sykes and Sturzaker (n 157).

174 King Charles III, ‘The King’s Speech 2024’ (UK Parliament, 17 July 2024) <www.gov.uk/government/speeches/the-kings-speech-2024>.

175 Gu Qing Yang and RN Sugitha Nadarajah, ‘A Visionary’s Experience in Incorporating Infrastructure into Long-Term Urban Planning—Interview with Dr. Liu Thai Ker’ (2017) 1 Journal of Infrastructure, Policy and Development 90, 93.

